# The Impact of Surgery in IDH 1 Wild Type Glioblastoma in Relation With the MGMT Deregulation

**DOI:** 10.3389/fonc.2019.01569

**Published:** 2020-01-24

**Authors:** Francesco Marchi, Nora Sahnane, Roberta Cerutti, Debora Cipriani, Jessica Barizzi, Federico Mattia Stefanini, Samantha Epistolio, Michele Cerati, Sergio Balbi, Luca Mazzucchelli, Fausto Sessa, Gianfranco Angelo Pesce, Michael Reinert, Milo Frattini

**Affiliations:** ^1^Service of Neurosurgery, Neurocenter of the Southern Switzerland, Regional Hospital of Lugano, Lugano, Switzerland; ^2^Unit of Pathology, Department of Medicine and Surgery, University of Insubria-ASST Sette Laghi, Varese, Italy; ^3^Institute of Pathology, Locarno, Switzerland; ^4^Department of Statistics, Computer Science, Applications, University of Florence, Florence, Italy; ^5^Division of Neurological Surgery, Department of Biotechnology and Life Sciences, University of Insubria-ASST Sette Laghi, Varese, Italy; ^6^Radiation Oncology, Oncology Institute of Southern Switzerland, Bellinzona, Switzerland; ^7^Faculty of Medicine, University of Bern, Bern, Switzerland

**Keywords:** glioblastoma, extent of resection, MGMT, overall survival, progression free survival, temozolomide

## Abstract

**Object:** The treatment of choice in glioblastoma (GBM) is the maximal surgical extent of resection (EOR) followed by adjuvant chemo-radiotherapy. Furthermore, methylguanine-DNA methyltransferase (MGMT) promoter methylation is associated with prolonged overall survival (OS) and progression free survival (PFS). The objective of the present study is correlate the biomolecular aspects in relation with EOR.

**Materials and methods:** We analyzed a series of 116 patients with IDH-1 wild type GBM and different EOR (Gross Total Resection—GTR-, Partial Resection—PR- and Biopsy), treated with adjuvant chemo-radiotherapy. The MGMT status was analyzed in terms of promoter methylation and protein expression.

**Results:** When GTR was possible, OS and PFS were significantly better compared to the other two groups (*p* = 0.001 and *p* = 0.035, respectively). MGMT methylation was significantly associated with better OS in the biopsy group (*p* = 0.022) and better OS and PFS in PR (*p* = 0.02 and *p* = 0.012, respectively), but not in the GTR group (*p* = 0.252 for OS, *p* = 0.256 for PFS) nor the PFS in the biopsy group (*p* = 0.259). MGMT protein expression levels do not show any association with OS and PFS, regardless of the type of surgery.

**Conclusions:** Our study confirms the positive association of a safe maximal EOR with better OS and PFS, and indicates a positive prognostic value of MGMT methylation status only in case of the presence of residual tumor tissue. MGMT protein expression seems not to play a clinical role in relation with the type of surgery.

## Introduction

Glioblastoma (GBM) is the most common primary malignant brain tumor in adults (1). Currently, safe optimal surgical resection followed by adjuvant radiotherapy and chemotherapy is considered as the standard treatment approach for patients with GBM (2–4). However, despite advances in the last three decades and aggressive multimodal treatment, outcome remains poor for patients with GBM, with a median overall survival of 14–17 months from time at diagnosis (2, 3). Many studies have reported a positive correlation between the extent of resection (EOR) and the overall survival (OS) in patients with GBM, in particular for patients undergoing Gross Total Resection (GTR) with respect to whom receiving only a Subtotal Tumor Resection (STR) (5).

When GTR is not possible (due to several causes such as disease location and extension, general conditions of the patient), no clearly recognized criteria are proposed in the literature in order to stratify the STR group and, as a consequence, the threshold of EOR required for better prognosis remains controversial. Moreover, a recent observational retrospective study (6) has enrolled 38 patients who underwent PR and 78 biopsies and has pointed out that PR failed to improve OS and PFS compared with biopsy in patients with GBM (*p* = 0.84 and 0.48, respectively). Even the propensity score matching (PSM) between the PR and biopsy groups, according with this study, did not show any significant difference in OS and PFS between the groups (*p* = 0.51 and 0.75, respectively). The hazard ratios for OS and PFS of PR compared with biopsy were 0.98 and 0.73, respectively; however, the difference was not statistically significant (*p* = 0.96 and 0.39, respectively). Moreover, the surgical complication rate was higher in the PR group (14/32, 43.7%) than in the biopsy group (9/78, 11.5%) (*p* < 0.01). The cited study confirms that no significant association and benefit has been clearly yet demonstrated between the different degrees of PR and the biomolecular markers in regards of OS and PFS.

Methylguanine-DNA methyltransferase (MGMT) plays the pivotal role in the management of GBM patients: hypermethylation of MGMT promoter (causing absence of MGMT protein expression) leads to a higher response to temozolomide (TMZ), thus improving the patients' outcome (2, 7, 8). Furthermore, it has been discovered that additional mechanisms may decrease the MGMT expression. Approximately 20% of all patients with unmethylated GBM experiences an unexpected favorable outcome after chemoradiation, because mRNA expression was found to be unexpectedly low (9–11).

Only a few studies have investigated the influence of surgery on the clinical outcome in regards of the molecular markers (4, 12). Gessler et al. in their recent publication confirm that GTR is able to prolong PFS and OS when compared to incomplete resection, and the presence of methylation is a prognostic factor increasing significantly PFS and OS (4).

The aim of this study is to assess the relation between EOR and MGMT status (in terms of MGMT deregulation methylation and protein expression) by analyzing the clinical outcome (PFS and OS) of radio-chemotherapy treated IDH-1 wild type GBM patients, in correlation with the type of surgery.

## Materials and Methods

This bi-center retrospective cohort study included patients with newly diagnosed histologically reviewed GBM with IDH-1 wild type status from 2004 until 2013.

This work has been conducted in compliance with the protocol, the current version of the Declaration of Helsinki, the ICH-GCP or ISO EN 14155 (as far as applicable) as well as all national legal and regulatory requirements. Data and samples have been collected and analyzed for the study purpose only after the required authorizations from the competent Ethics Committees were obtained (Rif. CE 3086-2016-01108).

Inclusion criteria consist of age >18 years, histological diagnosis of IDH-1 wild type GBM (WHO IV), therapy with TMZ according with the Stupp scheme (60 Gray radiotherapy and concomitant chemotherapy with TMZ, followed by six cycles of maintenance TMZ), death caused by GBM, tissue availability for biomolecular analyses.

The OS (defined as the time from surgery to the date of death) and PFS (defined as the time from the first radio-chemotherapy treatment to the date of clinical or radiological progression according with the RANO criteria) were analyzed. Regarding the type of surgery, three groups were defined according with the post-op MRI performed in the first 72 h: GTR (with no contrast-enhancing residual tissue visible on T1 injected MRI sequences), incomplete Partial Resection (PR) (with evidence of contrast-enhancing residual tumor) and Biopsy.

### Molecular Analyses

#### MGMT Promoter Methylation

Tissues for genomic DNA isolation were dissected manually from three 8-μm sections and DNA was obtained using automatic extraction (Maxwell, Promega, Madison, WI, USA). About 50–100 ng of DNA were subjected to bisulphite treatment using EZ DNA Methylation-Gold TM kit (Zymo Research, Irvine, CA, USA). Methylation status of six consecutive cytosines of MGMT promoter (chr10:131,265,507−131,265,556) was assessed by PCR-pyrosequencing of bisulphite-treated DNA by using MGMT Plus kit according to the recommended protocol (Diatech Pharmacogenetics, Jesi, Italy). A cut-off of 10% was set to score presence of promoter methylation. This value was determined calculating the limit of negative controls (DNA samples from 15 FFPE healthy brain tissues) for each cytosine (mean of methylation ratio adding 2 × the Standard Deviation) assuming a Gaussian distribution of the raw signal from negative samples. The limit corresponded to 95% of the observed negative values.

#### MGMT Immunohistochemistry

Immunohistochemical reactions for MGMT protein were performed on whole tissue sections obtained from formalin-fixed, paraffin-embedded (FFPE) tumor blocks. Three -μm-thick sections were deparaffinized, rehydrated and pretreated with citrate buffer pH6 in microwave oven for 20 min. Monoclonal primary antibody anti-MGMT, clone MT3.1 (Chemicon International, Temecula, CA, USA) was used at a dilution of 1/400 and applied overnight at 4°C, followed by a polymeric detection system (Ultravision DAB Detection System, LabVision, Fremont, CA, USA) according to the manufacturer's protocol. According to the literature, immunohistochemical positivity was scored when more than 5% of neoplastic cells showed an intense nuclear staining (13, 14).

### Statistical Analyses

Mean and median values were calculated at first to summarize results of each variable. The relative chi-square values were calculated on pairs of variables to describe the statistical association existing among variables: null hypothesis stating the lack of marginal association between pairs of variables (after discretization) was assessed through the chi-square test.

OS and PFS curves for censored data were obtained using the Kaplan-Meier estimator; comparisons of curves given different molecular characterizations were performed by logrank tests. PFS curves were also estimated and tested within strata defined by the variable of surgery.

All the analyses, graphs and reports were performed using the R software [R] and the following R packages: survival, bootstrap, rmarkdown, knitr (15–18).

## Results

The study includes 116 patients, 57 females (49.1%) and 59 males (50.9%). Among them, 81 underwent GTR of the tumor (69.8%), 18 PR (15.5%) while in 17 cases only biopsy was performed (14.7%). In 92 patients (corresponding to 79.3% of the whole cohort), we observed progression of the disease (PD), while the remaining 24 cases (20.7%) include both the six patients who are still alive (*N* = 6) and the 18 patients who deceased for other causes with no evidence of tumor progression.

Concerning the biomolecular aspects, 71 samples showed absence of MGMT promoter methylation tumors (61.2%), 41 methylation (35.3%) while in four samples the methylation status was not evaluable (3.4%). The immunohistochemical evaluation of the MGMT protein revealed a positive expression in 54 samples (46.5%) and a negative expression in 44 cases (37.9%), while in 18 cases (15.5%) the assay did not give evaluable results (Table 1). As for clinical data, OS was 15.5 months and PFS was 7 months.

**Table 1:** Synoptic overview of the patient population.

**Patients**	**116**	
IDH1 wild type GBM WHO IV	116	
Female	57	49.1%
Male	59	50.9%
GTR	81	69.8%
PR	18	15.5%
Biopsy	17	14.7%
Surgery + Radio-chemotherapy	116	
Deceased	110	94.8%
Alive	6	5.1%
Progression Disease (PD)	92	79.3%
Alive with no evidence of PD	6	5.1%
Deceased with no evidence of PD	18	15.5%
MGMT methylated	41	35.3%
MGMT non-methylated	71	61.2%
MGMT non definable	4	3.4%
MGMT protein expression positive	54	46.5%
MGMT protein expression negative	44	37.9%
MGMT expression not evaluable	18	15.5%

*GBM, Glioblastoma; GTR, Gross Total Resection; MGMT, methylguanine-DNA methyltransferase; PR, Partial Resection*.

**Figure 1:**
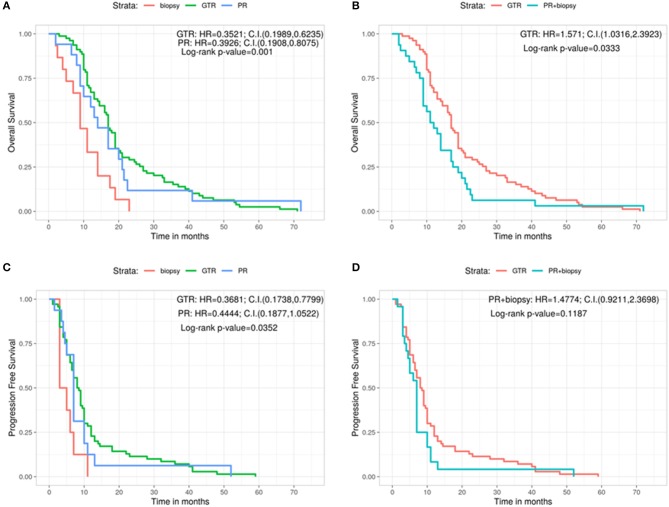
Overall survival for each type of surgery **(A)** and grouping PR and biopsy groups **(B)**. Progression free survival for each type of surgery **(C)** and grouping PR and biopsy groups **(D)**. C.I., Confidence interval; GTR, gross total resection; H.R., hazard ratio; PR, partial resection.

We then analyzed the correlation between the type of surgery and the OS (Figures 1A,B). Patients who underwent a GTR had a significantly better OS (17 months) compared with those in whom a PR (14 months) or a biopsy (9 months) had been performed (Log-rank *p* = 0.001; GTR: HR = 0.3521, 95%CI: 0.1989, 0.6235; PR: HR = 0.3926, 95%CI: 0.1908, 0.8075) (Figure 1A). Analogously, grouping the patients who sustained PR and biopsy, we confirmed a significant longer OS for patients subjected to GTR (GTR = 17 months, PR + Biopsy = 11.5 months, Log-rank *p* = 0.0333; GTR: HR = 1.571, 95%CI: 1.0316, 2.3923) (Figure 1B). Similar results were obtained by comparing the type of surgery and PFS (Figures 1C,D). Indeed, the GTR group presented a longer PFS compared with the PR group and the biopsy group (GTR = 8.25 months, PR = 7.50 months, Biopsy = 4.00 months, Log-rank *p* = 0.0352; GTR: HR = 0.3681, 95%CI: 0.1738, 0.7799; PR: HR = 0.4444, 95%CI: 0.1877, 1.0522) (Figure 1C). On the contrary, GTR did not give a significant greater PFS compared with the value of the other two groups considered together (GTR = 8.25 months, PR + Biopsy = 7.00, Log-rank *p* = 0.1187; PR + biopsy: HR = 1.4774, 95%CI: 0.9211, 2.3698) (Figure 1D).

**Figure 2:**
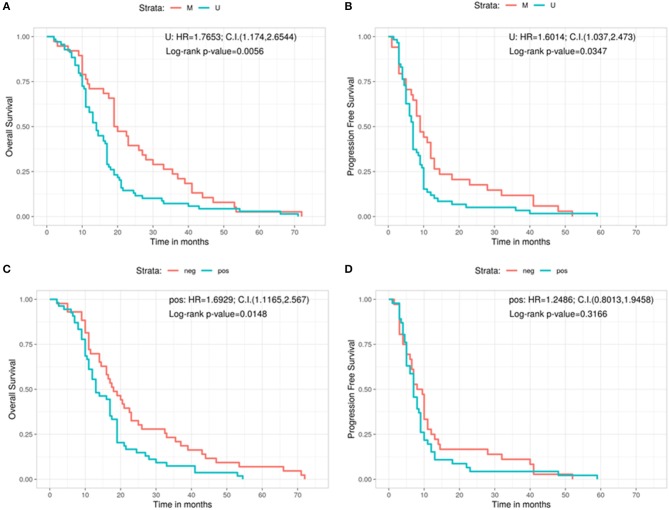
Overall survival **(A)** and progression free survival **(B)** according with the MGMT methylation status. Overall survival **(C)** and progression free survival **(D)** according with the immunohistochemistry results. C.I., Confidence interval; H.R., hazard ratio; M, methylated; neg, IHC negative; pos, IHC positive; U, unmethylated.

Afterwards, the OS and the PFS were analyzed in relation with the MGMT promoter methylation and the protein expression evaluated by immunohistochemistry (IHC) (Figures 2A–D). The OS is significantly better in MGMT methylated GBMs than in MGMT unmethylathed ones (methylated: 19.5 months, unmethylathed: 14 months, Log-rank *p* = 0.0056; U: HR = 1.7653, 95%CI: 1.174, 2.6544) (Figure 2A). Same positive correlation, statistically significant, was found for PFS, with 9 months before progression in MGMT methylated patients and 7 months for MGMT unmethylated ones (Log-rank *p* = 0.0347; U: HR = 1.6014, 95%CI: 1.037, 2.473) (Figure 2B).

Regarding the levels of MGMT protein expression, patients who had low MGMT protein expression had a significantly improved OS compared with patients who had high MGMT protein expression (18 vs. 13 months; Log-rank *p* = 0.0148; Pos: HR = 1.6929, 95%CI: 1.1165, 2.567) (Figure 2C). On the contrary, no significant correlation was observed for MGMT protein expression in regards of PFS (8.75 months for GBMs showing low protein expression and 7 months for those with high protein expression, Log-rank *p* = 0.3166; Pos: HR = 1.2486, 95%CI: 0.8013, 1.9458) (Figure 2D).

**Figure 3:**
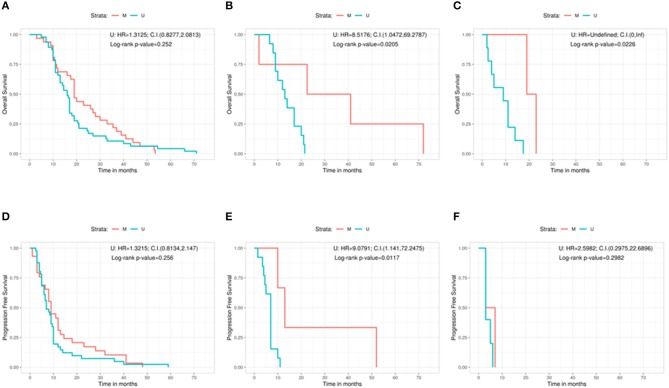
Overall survival according with the MGMT methylation status for the gross total resection group **(A)**, the partial resection group **(B)**, and the biopsy group **(C)**. Progression free survival according with the MGMT methylation status for the gross total resection group **(D)**, the partial resection group **(E)**, and the biopsy group **(F)**. C.I., Confidence interval; H.R., hazard ratio; M, methylated; U, unmethylated.

Furthermore, we analyzed the outcome (in terms of both OS and PFS) subdividing the cohort on the basis of the three different types of surgery, in relation with the methylation status of the MGMT gene (Figures 3A–F). No significant correlation was found in patients with GTR between OS and methylation status (methylated = 19 months, unmethylated = 16 months, Log-rank *p* = 0.252; U: HR = 1.3125, 95%CI: 0.8277, 2.0813) (Figure 3A). A positive correlation was shown, instead, in the PR group, in which MGMT methylated patients had a better OS compared with the unmethylated ones (methylated = 31.8 months, unmethylated = 13.0 months, Log-rank *p* = 0.0205; U: HR = 8.5176, 95%CI: 1.0472, 69.2787) (Figure 3B). The same positive statistically significant correlation was observed in patients who underwent biopsy (methylated = 21 months, unmethylated = 9 months, Log-rank *p* = 0.0226; U: HR = undefined, 95%CI: 0, ∞) (Figure 3C).

As regards the PFS, patients who underwent a GTR and were MGMT methylated did not show a better outcome if compared with patients carrying MGMT unmethylated GBM (methylated = 9 months, unmethylated = 7 months, Log-rank *p* = 0.256; U: HR = 1.3215, 95%CI: 0.8134, 2.147) (Figure 3D). On the contrary, a statistically significant better PFS was noted in MGMT methylated patient with respect to MGMT unmethylated ones in the PR group (methylated = 13 months, unmethylated = 7 months, Log-rank *p* = 0.0117; U: HR = 9.0791, 95%CI: 1.141, 72.2475) (Figure 3E). Finally, absence of correlation between PFS and the methylation status was observed in the group of patients who underwent a biopsy (methylated = 5 months, unmethylated = 3 months, Log-rank *p* = 0.2982; U: HR = 2.5982, 95%CI: 0.2975, 22.6896) (Figure 3F).

**Figure 4:**
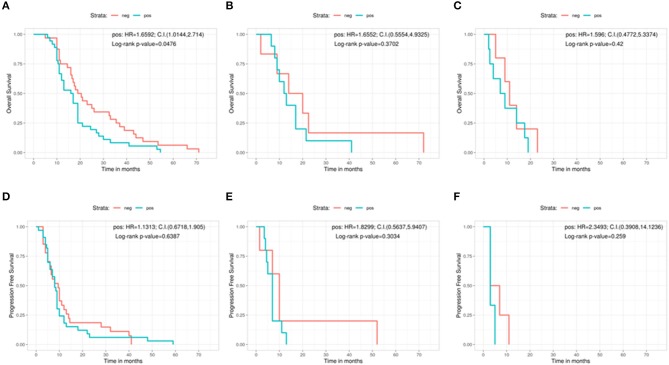
Overall survival according with the protein expression in immunohistochemistry for the gross total resection group **(A)**, the partial resection group **(B)**, and the biopsy group **(C)**. Progression free survival according with the protein expression in immunohistochemistry for the gross total resection group **(D)**, the partial resection group **(E)**, and the biopsy group **(F)**. C.I., Confidence interval; H.R., hazard ratio; neg, IHC negative; pos, IHC positive.

Moreover, we analyzed the same variables (clinical outcome and EOR) on the light of the results of MGMT protein expression (Figures 4A–F). In terms of OS, patients who underwent a GTR with a low protein expression had a significant better outcome with respect to patients with high MGMT expression (low protein expression = 19.8 months, high protein expression = 16.5 months, Log-rank *p* = 0.0476; Pos: HR = 1.6592, 95%CI: 1.0144, 2.714) (Figure 4A). On the contrary, no significant correlations were found in the PR and in the biopsy groups regarding MGMT protein expression in terms of OS: 17 months for low protein expression patients vs. 12.5 months for those with high protein expression in the PR group (Log-rank *p* = 0.3702; Pos: HR = 1.6552, 95%CI: 0.5554, 4.9325) (Figure 4B), and 11 months in low protein expression patients vs. 8 months in patients with high MGMT expression in the biopsy group (Log-rank *p* = 0.42; Pos: HR = 1.596, 95%CI: 0.4772, 5.3374) (Figure 4C).

As regards PFS, IHC showed no significant relation between low and high protein expression patients in any group. In the GTR patients, the difference in PFS between low expressed and high expressed cases was 9.5 vs. 8 months (Log-rank *p* = 0.6387; Pos: HR = 1.1313, 95%CI: 0.6718, 1.905) (Figure 4D). In the PR group, PFS was 10 months for low protein expression patients vs. 7 months for patients with high MGMT protein levels (Log-rank *p* = 0.3034; Pos: HR = 1.8299, 95%CI: 0.5637, 5.9407) (Figure 4E). In the biopsy group, patients showing low protein expression level had a PFS of 5 months compared with 3 months of those with a high MGMT protein expression (Log-rank *p* = 0.259; Pos: HR = 2.3493, 95%CI: 0.3908, 14.1236) (Figure 4F).

## Discussion

Our paper presents a bicentric, retrospective study including a series of patients affected by IDH-1 wild type GBM treated with chemotherapy and radiotherapy after surgery.

Firstly, compared to the data in the literature, we tried to define three new unambiguous categories of surgical treatment: GTR, when no evidence of residual tumor on the T1 injected post-op sequences MRI; PR, if any enhancement is visible (independently of the residual volume); and biopsy, if only a small piece of tumor is taken for analysis. We think that this categorization is a novelty in literature considering the PR as the presence of residual tumor, regardless its volume. All the other studies, in fact, have defined the residual volume in a percentage that, in the majority of cases, may be subjective.

Our results confirm the well established statement for which, when feasible, GTR is the gold standard to achieve in the surgical treatment of GBMs with a longer term OS in this group of patients vs. both the PR group and the biopsy group (17 vs. 14 vs. 9 months for the different groups, respectively). Similar data are obtained comparing the GTR group with the PR + biopsy combined one (17 vs. 11.5, respectively). However, as stated above, the GTR in our work is the complete absence of residual tumor on the postop MRI and not, as in literature, the variable majority of tumor resected (>95 or >97%) considering the difficulty in the objective calculation of the percentage of the remnant tumor.

Regarding the PFS, we observed the same significant positive relation in favor of the GTR group compared with the PR and biopsy groups taken singularly (8.25 vs. 7.5 vs. 4, respectively) but not when the latter two (PR + biopsy) are assembled together. Also these data confirm the already published ones but, differently, in our work, the PR is considered any enhancement of any size visible on the postoperative MRI.

In respect of the MGMT methylated status and the better outcome, our results are in line with the main series published in literature (7, 19) with a median OS of 19.5 months for patients with a MGMT methylated GBM vs. 14 months for patients with an MGMT unmethylated GBM and 2 months more of PFS between the two groups. However, while the better prognosis in terms of OS was confirmed by protein expression levels assessed by IHC, no significant correlation between the two groups (low and high MGMT protein expression) was shown for the PFS with this analysis. Therefore, our study confirms the lower diagnostic value of IHC as compared to the evaluation of the MGMT methylation status.

The most interesting results have been obtained matching the MGMT status and the EOR. While we observed that the MGMT status is positively and significantly correlated to the clinical outcome in the PR group (OS of 31.8 months for patients with MGMT methylated GBM with respect to only 13 months for MGMT unmethylated GBM, PFS of 13 vs. 7 months, respectively), in the group of patients who underwent a GTR we did not observe any significant association between OS or PFS and the methylation status of MGMT gene. Likewise, the simple biopsy did not change significantly the outcome in terms of PFS in methylated vs. unmethylated patients with only 2 months achieved before disease progression. However, in terms of OS, patients characterized by an MGMT methylated GBM have an advantage of 12 months with respect to patients with an MGMT unmethylated tumor. Our results confirm those recently published but using clearly, objective and widely applicable categories of EOR (4, 12). Therefore, we can postulate that the identification of MGMT promoter methylation may identify a group of GBM patients who are correlated with a better response to the combined chemo-radiotherapy treatment only if the neoplastic tissue is still present. On the contrary, patients who are *bona fide* radically resected, will experience the same follow-up with respect to the combined chemo-radiation therapy.

IHC represents the classic worldwide used method for the detection of protein expression. However, especially for MGMT protein (as for other proteins located in cell nucleus), it can be sometimes hard to be evaluated, leading to the conclusion that MGMT promoter methylation should be the preferred method for assessing MGMT deregulation instead of IHC. In fact, literature reports that sometimes there is no correlation between MGMT expression and its promoter methylation (20). Also in our series IHC does not confirm the expected significant correlation between low protein expression and better clinical outcome in PR and biopsy groups. Indeed, the only favorable significant correlation (borderline, *p* = 0.047) for the low protein expression was noted in the OS for the patients who underwent GTR: they presented almost 20 months of survival vs. 16.5 months of those with samples expressing high levels of MGMT protein. Neither the GTR nor the PR or the biopsy group showed a significant relation between IHC and PFS.

Beside IHC limits, also the definition of MGMT promoter methylation is sometimes challenging, however literature reports some cut-off values that can be used to define a sample as positive for MGMT methylation (21). In our work we applied a cut-off of 10% that was decided on the bases of an internal control evaluation of a cohort of negative (healthy tissues) samples.

Our study present some limitations, such as the number of patients included in the analysis is small if compared to other series, even if not inferior to the majority of studies published. For this reason, the results of the present work are in line with those in the literature and seem to be not innovative. However, several aspects are not exhaustively treated in literature and some features can be helpful in everyday practice as, for example, the simple classification between GTR and PR or the importance of radio-chemotherapy in case of residual enhancement on the postoperative images.

To conclude, the present study confirms the better outcome in patients with GBM who sustained a GTR: maximal EOR in surgery seems to be confirmed as the most important prognostic value for OS and PFS in the treatment of GBM patients, thus indicating that, whenever possible, this is the goal that must be pursued by clinicians. Under these conditions, the most relevant biomarker, MGMT, does not seem to play any prognostic role. Theoretically and provocatively, the present study states that chemo-radiotherapy, in presence of complete resection, could not influence significantly OS and PFS, playing a substantial role only in case of residual tumor. When surgery is not possible, the MGMT methylated status is proven to be a favorable marker for OS and PFS in patients with remnant tumor after an incomplete tumor resection (in both PR and biopsied patients). On the contrary, IHC expression does not correlate with different OS or PFS in relation with the type of surgery, thus confirming the discrepancy between protein expression and MGMT methylation status evaluation and suggesting a superior predictive role of the latter.

We think that the current study, to the best of our knowledge, is one of the few studies which correlates biological aspects and different type of surgery in GBM patients treated with a combined chemo-radiotherapy. However, considering the continuous changes in the field of brain tumors, further studies are needed in order to confirm our data and to identify other possible correlations between the newest biological markers, the clinical outcome and the surgical treatment in patients with GBM.

## Data Availability Statement

The raw data supporting the conclusions of this article will be made available by the authors, without undue reservation, to any qualified researcher.

## Ethics Statement

This work has been conducted in compliance with the protocol, the current version of the Declaration of Helsinki, the ICH-GCP or ISO EN 14155 (as far as applicable) as well as all national legal and regulatory requirements. Data and samples have been collected and analyzed for the study purpose only after the required authorizations from the competent Ethics Committees were obtained (Rif. CE 3086-2016-01108).

## Author Contributions

FM and NS collected all clinical data and wrote the first draft of the manuscript. FM, DC, FS, GP, MR, and MF selected the cohort for the analyses. JB, MC, LM, FS, and GP selected all the specimens at histologic level. NS, RC, SE, and SB performed all the molecular characterization. SE, RC, and MF evaluated MGMT results. JB, MC, LM, FS, and GP evaluated IHC experiments. FMS was responsible for all the statistical analyses. FM, NS, and SE prepared the final version of the manuscript. FM, FS, GP, MR, and MF supervised the whole project.

### Conflict of Interest

The authors declare that the research was conducted in the absence of any commercial or financial relationships that could be construed as a potential conflict of interest.

## Abbreviations

EOR: Extent of Resection
GBM: Glioblastoma
GTR: Gross Total Resection
IDH: Isocitrate Dehydrogenase
IHC: immunohistochemistry
MGMT: methylguanine-DNA methyltransferase
PFS: Progression Free Survival
PR: Partial Resection
OS: Overall Survival
STR: Subtotal Resection
TMZ: temozolomide.
